# Clinical Assessment of Automated Non-Contact Tonometer: Interchangeability with Goldmann Applanation Tonometry and Repeatability

**DOI:** 10.3390/jcm14082726

**Published:** 2025-04-15

**Authors:** Michael Chaglasian, Huiyuan Hou, Mayra Tafreshi, Mary K. Durbin, Ece Turhal, David Kasanoff, Sasan Moghimi, Alex S. Huang

**Affiliations:** 1 Illinois College of Optometry, Chicago, IL 60616, USA; mchaglas@ico.edu; 2 Topcon Healthcare Inc., Oakland, NJ 07436, USA; mtafreshi@topcon.com (M.T.); mdurbin@topcon.com (M.K.D.); eturhal@topcon.com (E.T.); 3 New View Optometric Center, La Mesa, CA 91942, USA; davksnf@gmail.com; 4 Hamilton Glaucoma Center and Shiley Eye Institute, Viterbi Family Department of Ophthalmology, University of California, San Diego, CA 92093, USA; sasanimii@yahoo.com (S.M.); aahuang@health.ucsd.edu (A.S.H.)

**Keywords:** intraocular pressure, tonometer, interchangeability, agreement, repeatability

## Abstract

**Background/Objectives**: This study aimed to evaluate the clinical interchangeability of intraocular pressure (IOP) measurements between a non-contact tonometer (NCT), the TRK-3 OMNIA, and Goldmann applanation tonometry (GAT) and to assess the repeatability of TRK-3 measurements. **Methods**: This prospective, multicenter study included 120 subjects stratified into three IOP groups based on GAT measurements: low IOP (7–16 mmHg), intermediate IOP (>16 to <23 mmHg), and high IOP (≥23 mmHg). The study eye was randomly selected from each subject. IOP was measured using both TRK-3 OMNIA and GAT following a standardized protocol. Agreement between the two methods was evaluated using Bland–Altman analysis, 95% limits of agreement (LoA), and equivalence testing via the two one-sided test (TOST) approach with a predefined ±5 mmHg margin. Linear regression analysis was performed to characterize the relationship between TRK-3 and GAT measurements. The repeatability of TRK-3 measurements was assessed using the intraclass correlation coefficient (ICC), repeatability limit, and coefficient of variation (CV). **Results**: Across all subjects, the mean difference between TRK-3 OMNIA and GAT IOP measurements was −0.2 mmHg. TRK-3 OMNIA overestimated IOP in the low IOP group (mean difference: 2.1 mmHg, LoA: −1.2 to 5.4 mmHg) and underestimated in the high IOP group (mean difference: −2.4 mmHg, LoA: −5.9 to 1.1 mmHg), while agreement was highest in the intermediate IOP group (−0.2 mmHg, LoA: −2.9 to 2.5 mmHg). Despite the systematic trend, equivalence testing confirmed statistical equivalence across all groups, with 90% confidence intervals (CI) of 1.7 to 2.5 mmHg (low IOP group), −0.6 to 0.2 mmHg (intermediate IOP group), and −2.9 to −2.0 mmHg (high IOP group). Linear regression analysis found a strong correlation (r = 0.92) between TRK-3 and GAT. The TRK-3 OMNIA demonstrated good repeatability, with an ICC of 0.94, a repeatability limit of 3.12 mmHg, and a CV of 5.65%. The repeatability limits were 2.22 mmHg, 2.60 mmHg, and 4.19 mmHg in the low, intermediate, and high IOP groups, respectively. **Conclusions**: TRK-3 OMNIA and GAT measurements showed strong agreement, statistical equivalence, and a high correlation, supporting their clinical interchangeability. TRK-3 also demonstrated high repeatability. These findings indicate that this automated non-contact tonometer provides reliable and repeatable IOP measurements, supporting its integration into routine clinical workflows.

## 1. Introduction

The measurement of intraocular pressure (IOP) is essential for diagnosing and managing glaucoma and other ocular conditions [1]. IOP is the only modifiable risk factor for glaucoma, a leading cause of irreversible blindness worldwide, and its reduction remains the only proven strategy to slow glaucoma progression. Therefore, frequent and accurate IOP assessment is critical in glaucoma management [2,3,4,5,6].

Various tonometry methods are employed in clinical practice, each with its own advantages and limitations. Among them, Goldmann applanation tonometry (GAT) is widely regarded as the gold standard and serves as the basis for numerous clinical guidelines and protocols [7]. However, GAT has several drawbacks associated with its clinical use. It requires direct corneal contact, necessitating topical anesthesia and fluorescein dye, which may cause discomfort and carries risks of corneal abrasion and infection transmission. Additionally, this technique demands a skilled examiner due to its steep learning curve. Moreover, GAT is unsuitable for patients in a supine position or those unable to cooperate with slit lamp examinations. Its lack of portability and reliance on a slit lamp setup further limit its feasibility for large-scale population screening, although portable applanation devices such as the Perkins tonometer can partially address this limitation [7,8].

To address some of the limitations of GAT, several alternative tools for IOP measurement have been developed to complement or, in certain cases, replace GAT. Rebound tonometry (RBT) is one such method that has gained popularity due to its portability and ease of use, particularly in pediatric and home-monitoring applications [9]. In addition to RBT, non-contact tonometers (NCTs) are widely used in clinical practice [8,10]. By utilizing an air puff to applanate the cornea, NCTs eliminate the need for corneal contact and topical anesthesia, making them more comfortable and safer for patients. They are user-friendly and suitable for mass screenings and situations where rapid IOP assessment is desired [8,10]. Since their introduction in the 1970s, NCTs have evolved significantly, providing a practical approach for IOP measurement [11]. Advancements in sensor technology and calibration algorithms have improved the accuracy of NCT measurements, extending the use of IOP measurements beyond clinical management to routine screenings and large-scale population studies. Currently, commercially available NCTs range from standalone devices, such as the Topcon CT-80, Reichert 7CR, and Canon TX series, to multi-functional platforms like the Topcon CT-1P, Topcon TRK-2P, and Nidek Tonoref series, which integrate tonometry with additional functions like keratometry, pachymetry, and refractometry. These multi-functional devices enhance clinical efficiency by consolidating multiple ocular measurements into a single system, streamlining workflow, and reducing patient burden.

Clinicians frequently encounter situations where NCTs are more accessible or practical, yet clinical decisions are traditionally based on GAT-derived IOP values. Moreover, inconsistent use of these methods during follow-up may lead to variability in recorded IOP values, potentially impacting disease monitoring and management. If the relationship between these measurements is not well understood, discrepancies between methods could lead to a misinterpretation of the disease status or inappropriate clinical decisions [8,11,12]. Establishing the clinical interchangeability between NCT and GAT measurements is therefore essential [8,11,12], as it allows for evidence-based application in practice. NCT values may serve as a direct reference, or conversion to GAT-equivalent values may be preferred depending on clinical context and decision-making needs.

Another element of reliability is repeatability, which ensures that repeated measurements under identical conditions produce consistent results, reducing variability that could interfere with disease monitoring. This makes good repeatability a crucial requirement for a tonometer intended for routine clinical use [13,14].

The TRK-3 OMNIA, an auto kerato-refracto tonometer, integrates an automated non-contact tonometer, keratometer, refractometer, and pachymeter into a single device. It offers fully automated alignment and measurement without the need for manual adjustments. By automating the measurement of IOP, refractive errors, corneal curvature, and central corneal thickness within a single system, it enhances pretesting efficiency in clinical practice [15]. However, thorough evaluation is essential to ensure the appropriate clinical application of new technology and ultimately improve patient care. This study aims to evaluate the interchangeability between TRK-3 and gold standard GAT IOP measurements, as well as the repeatability of TRK-3 OMNIA IOP measurements, to aid clinicians in making informed decisions when utilizing this device in various clinical scenarios.

## 2. Materials and Methods

This prospective, randomized, multicenter study enrolled subjects who provided written informed consent. The study protocol was approved by the Advarra Institutional Review Board (6100 Merriweather Drive, Suite 600 Columbia, MD 21044. IRB Approval Pro00076158, dated 5 January 2024) and was conducted in accordance with the Declaration of Helsinki and the Health Insurance Portability and Accountability Act for research involving human subjects.

### 2.1. Study Population

Participants were recruited from 2 clinical sites in the United States—the New View Optometric Center (7339 El Cajon Blvd., Suite G, La Mesa, CA 91942, USA) and Illinois College of Optometry (3241 South Michigan Ave., Chicago, IL 60616, USA). This study enrolled 120 subjects, evenly stratified into three IOP groups based on GAT measurements: low IOP (7 to 16 mmHg), intermediate IOP (>16 to <23 mmHg), and high IOP (≥23 mmHg), following the subgroup definitions specified in standards for tonometer performance evaluation. Subjects underwent an ocular examination to determine eligibility for study enrollment. Demographic and clinical characteristics, including age, sex, and ocular history, were recorded at the time of enrollment. To determine eligibility, a measurement of preliminary IOP, corneal astigmatism, and/or central corneal thickness was performed if not available from historical data within 3 months.

Inclusion criteria required subjects to be at least 22 years old at the time of informed consent and to provide voluntary written consent for participation.

Exclusion criteria included individuals with only one functional eye; difficulty in ocular fixation or eccentric fixation; a history of corneal surgery; microphthalmia; buphthalmos; keratoconus; corneal or conjunctival lesions or infections; a central corneal thickness of less than 500 μm or greater than 600 μm; corneal astigmatism exceeding 3D; and known allergies to ophthalmic anesthetics or sodium fluorescein. Additionally, individuals who had used soft contact lenses within the last three months or hard contact lenses within the last six months; had dry eyes and were taking prescription medication or using artificial tears daily; or had blepharospasm or nystagmus were also excluded from this study.

The investigators followed a predefined randomization scheme to select the study eye. However, randomization could be overridden under specific conditions: if only one eye met the eligibility criteria, that eye was designated as the study eye.

### 2.2. Intraocular Pressure (IOP) Measurement

IOP was measured using both an NCT and GAT by licensed optometrists from the two participating clinical sites. All measurements followed standardized procedures that included two persons to ensure consistency across study sites and to enable masking to the highest extent possible: an operator who performed the measurement and was masked to the quantitative result and a recorder who noted the quantitative result. NCT was performed before GAT to minimize potential corneal alterations from the contact-based applanation method, ensuring a more consistent measurement.

**Figure 1:**
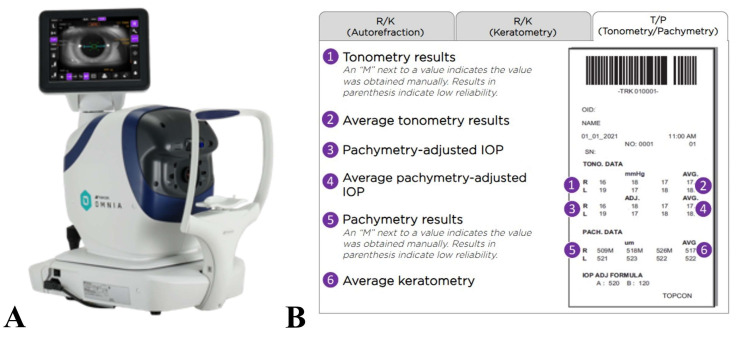
(**A**) TRK-3 OMNIA device. (**B**) Sample printout displaying tonometry and pachymetry results. The printout appearance may vary based on settings. In this example, three measurement sets are shown (default in multi-mode)., Pachymetry data and pachymetry-adjusted IOP values are included, though they were not used in this study.

For NCT, IOP was measured using the TRK-3 OMNIA auto kerato-refracto tonometer (Topcon Corporation, Tokyo, Japan) (Figure 1). The study eye underwent automatic triplicate measurements, with the device allowing up to four additional re-measurements to obtain a final set of three valid readings, if needed. The measurement process was masked to the operator and reviewed by an unmasked reader to reduce bias. The measurement time was recorded using the TRK-3 printout. If three valid measurements were not obtained within seven attempts, the subject proceeded with testing using GAT. If any measurement exceeded 30 mmHg, the device mode was switched to a higher range (60 mmHg), and the procedure was repeated accordingly.

For GAT, the same operator who performed the TRK-3 OMNIA measurement also conducted the IOP measurement using the GAT. The study eye was measured at least once following the standardized procedure. Re-measurement was permitted up to three times if the initial measurement was deemed invalid. Once a valid measurement was obtained, no further testing was performed. The measurement results and time were recorded by an unmasked GAT reader, who was different from the masked GAT measurer.

### 2.3. Statistical Analysis

In general, descriptive statistics (n, mean, standard deviation (SD), and median) were used to summarize continuous variables. Frequencies and percentages were used to summarize categorical variables.

Each study eye had three valid IOP measurements from the TRK-3 OMNIA tonometer. The statistical analysis was based on the mean IOP obtained from these three measurements.

The agreement between IOP measurements obtained using the TRK-3 OMNIA and GAT was assessed using Bland–Altman analysis. The mean difference (bias) between the two methods and the 95% limits of agreement (LoA) were calculated. A Bland–Altman plot was generated to visualize the distribution of differences across the range of IOP values.

The equivalence between IOP measurements obtained using TRK-3 OMNIA and GAT was evaluated using the two one-sided test (TOST) approach. The TOST approach evaluates whether the mean difference in IOP measurements between TRK-3 and GAT falls within the predefined equivalence margin of ±5 mmHg, which is a commonly accepted threshold in international tonometry standards, as specified by the ISO 8612:2009 [16], JIS T7312:2015 [17], and ANSI Z80.10-2014 [18] standard for tonometer equivalence testing. A paired *t*-test was conducted for both lower and upper equivalence bounds. In accordance with standard statistical methodology for equivalence testing, the TOST procedure involves conducting two one-sided t-tests at a significance level of 0.05 for each direction, which corresponds to constructing a 90% confidence interval (CI). If the 90% CI for the mean differences were fully contained within the ±5 mmHg range, equivalence was concluded. The analysis was also conducted to IOP subgroups to examine consistency in equivalence across different pressure ranges.

A linear regression model was developed to evaluate the relationship between TRK-3 OMNIA and GAT measurements. The regression values, including slope, intercept, and correlation coefficient, were used to characterize the association between the TRK-3 OMNIA and GAT.

The repeatability of TRK-3 OMNIA IOP measurements was assessed using three repeated measurements per eye. Intraclass correlation coefficients (ICCs) were computed to assess measurement consistency. A two-way mixed-effects model was used, with eyes as the random effect and repeated measurements as the fixed effect. For each eye, the variance of the three measurements was calculated, and the repeatability limit was determined as 2.77 × repeatability standard deviation (RSD), where RSD was the square root of the mean variance across eyes. The coefficient of variation (CV%) was calculated as (RSD/overall mean IOP) × 100 to quantify measurement variability relative to the mean IOP. Repeatability metrics were computed for the overall cohort and stratified by IOP group (low, intermediate, and high).

## 3. Results

This study included 120 eyes from 120 subjects, with 40 subjects in each IOP group based on GAT measurements. The mean age was 56.4 ± 13.9 years, with a median of 59 years. The mean age in the high IOP group was 62.2 ± 11.2 years, while it was 56.0 ± 13.6 years in the intermediate IOP group and 50.9 ± 14.7 years in the low IOP group. Similarly, age distribution showed a higher proportion of individuals over 60 years in the high IOP group (62.5%) compared to the intermediate (40.0%) and low IOP groups (35.0%). The gender distribution was similar across groups. Racial composition varied, with a predominance of Caucasian participants in the low (75.0%) and intermediate IOP groups (60.0%), while the high IOP group had a majority of African American participants (75.0%). The demographics of the study subjects are summarized in Table 1.

Among the 120 randomly selected study eyes, the distribution of left and right eyes was nearly balanced, with 50.8% (61/120) assigned to the right eye (OD) and 49.2% (59/120) to the left eye (OS). Each group contained a total of 40 eyes with valid results on both devices. The IOP measured by TRK-3 OMNIA and GAT for each group was summarized by device in Table 2.

**Figure 2:**
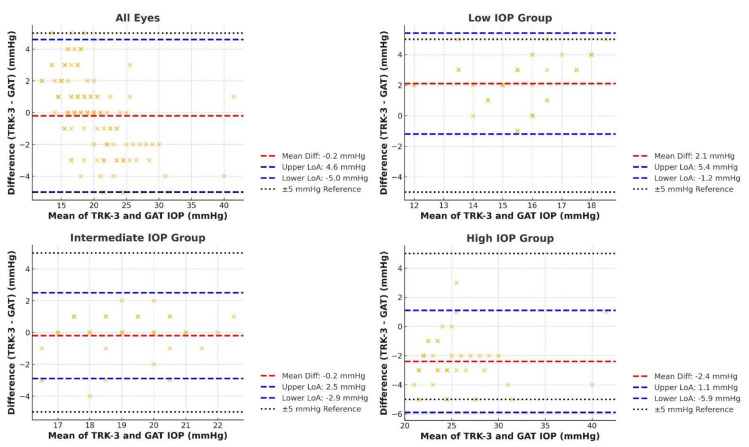
Bland–Altman plots of TRK-3 OMNIA and Goldmann applanation tonometry (GAT) intraocular pressure (IOP) measurements. Bland–Altman plots showing the agreement between TRK-3 OMNIA and GAT IOP measurements for all eyes and by IOP group (low, intermediate, and high). The red dashed line represents the mean difference, blue dashed lines indicate the limits of agreement (LoA, ±1.96 standard deviation), and black dotted lines mark the ±5 mmHg reference range.

The agreement between IOP measurements obtained using TRK-3 OMNIA and the GAT was assessed using the Bland–Altman method. Across all eyes, the mean difference (TRK-3 OMNIA–GAT) was −0.2 mmHg, with 95% LoA ranging from −5.0 to 4.6 mmHg. When stratified by IOP group, the low IOP group showed a mean difference of 2.1 mmHg (LoA: −1.2 to 5.4 mmHg), indicating that TRK-3 OMNIA tended to overestimate IOP compared to GAT in this range. The intermediate IOP group had a mean difference of −0.2 mmHg (LoA: −2.9 to 2.5 mmHg), suggesting a high agreement between TRK-3 OMNIA and GAT. In the high IOP group, TRK-3 underestimated IOP, with a mean difference of −2.4 mmHg (LoA: −5.9 to 1.1 mmHg). The results are shown in Figure 2.

To evaluate equivalence, a TOST was performed with an equivalence margin of ±5 mmHg. Across all eyes, the 90% CI (as per standard TOST methodology, i.e., two one-sided tests at α = 0.05) for the mean difference ranged from −0.6 to 0.2 mmHg, fully contained within the ±5 mmHg equivalence margin, confirming statistical equivalence. Similar findings were observed in all IOP subgroups, with 90% CIs of 1.7 to 2.5 mmHg in the low IOP group, −0.6 to 0.2 mmHg in the intermediate IOP group, and −2.9 to −2.0 mmHg in the high IOP group, all within the predefined equivalence range. Furthermore, when applying a stricter equivalence margin of ±3 mmHg, the 90% CI for the mean difference still remained within this narrower range across all eyes and IOP subgroups.

These results indicate that while TRK-3 OMNIA measurements exhibited a systematic bias in low and high IOP ranges, the differences remained within the equivalence margin that is considered clinically acceptable. The combination of Bland–Altman analysis and TOST confirms good agreement between the IOP measurements from TRK-3 OMNIA and GAT across the tested range.

A linear regression analysis was conducted to quantify the relationship between IOP measurements obtained using TRK-3 OMNIA and GAT. The regression equation with GAT as the dependent variable was

“GAT Result” = −3.79 + 1.20 × “TRK-3 Result”(1)

The intercept was −3.79 (95% CI −5.72, −1.86), and the slope was 1.20 (1.10, 1.29).

**Figure 3:**
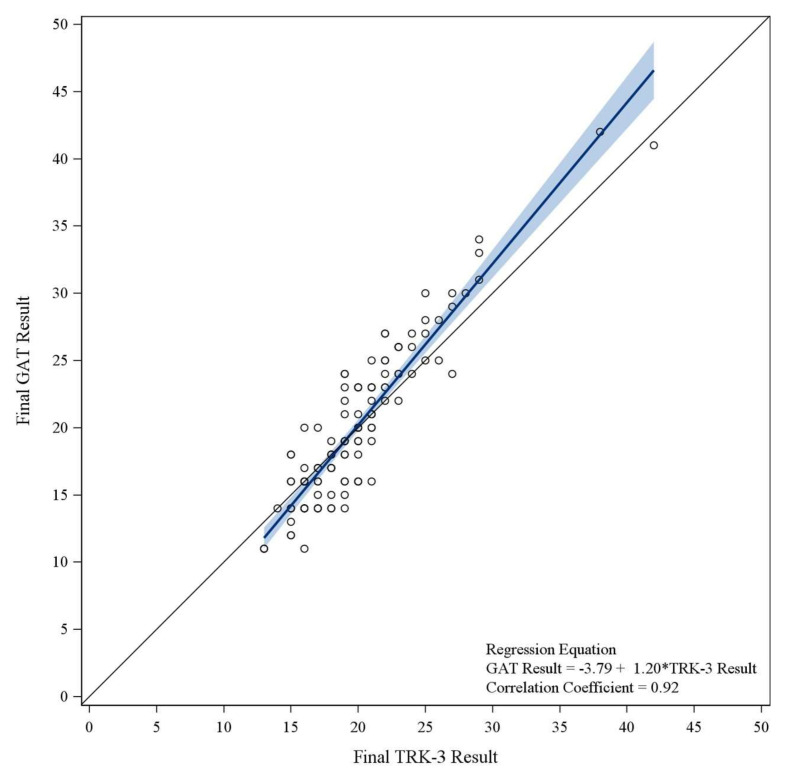
Linear regression analysis of TRK-3 OMNIA and Goldmann applanation tonometry (GAT) intraocular pressure (IOP) measurements. Scatter plot with linear regression lines showing the relationship between TRK-3 OMNIA and GAT IOP measurements. The regression equation and correlation coefficients are displayed, with the solid blue line representing the fitted regression model and the shaded area indicating the 95% confidence interval. The diagonal reference line (black) represents the line of identity (slope = 1).

The regression model showed a high correlation, with a correlation coefficient (r) of 0.92. The coefficient of determination (*R*^2^) was 0.84, indicating that 84% of the variance in one measurement can be explained by the other, which supports potential conversion between TRK-3 OMNIA and GAT measurements in clinical settings. The regression plot (Figure 3) illustrates the linear relationship between the two methods.

The repeatability of TRK-3 OMNIA IOP measurements is summarized in Table 3. The ICC was 0.94 overall, with values of 0.85, 0.79, and 0.90 in the low, intermediate, and high IOP groups, respectively. Overall, the repeatability limit was 3.12 mmHg, with the lowest in the low IOP group (2.22 mmHg) and the highest in the high IOP group (4.19 mmHg). The CV% was 5.65% overall, with slightly lower variability in the low and intermediate IOP groups (4.82% and 4.94%) and the numerically highest variability in the high IOP group (6.25%). These findings suggest that TRK-3 maintains good repeatability across all IOP levels.

## 4. Discussion

This study demonstrated that TRK-3 OMNIA provides IOP measurements that are consistent with GAT, with statistical equivalence confirmed across all IOP levels. TRK-3 also exhibited good repeatability. These findings indicate that TRK-3 delivers both accurate and repeatable IOP assessments, making it a reliable tool for clinical use and screening.

The increasing adoption of NCTs, particularly in settings where speed and patient comfort are prioritized, has led to growing interest in their clinical performance, including interchangeability with GAT and measurement reliability.

Understanding the agreement between NCTs and GAT remains critical for determining their role in the diagnosis and management of ocular conditions. Several studies have assessed the agreement between NCTs and GAT using Bland–Altman analysis to determine whether these non-contact devices can be used interchangeably with the gold standard. For instance, Patel et al. [11] evaluated multiple tonometers and found that the NCT (Topcon CT-80) yielded a mean difference of –0.006 mmHg with 95% LoA ranging from –4.7 to 4.6 mmHg [11]. In another study [19], a portable NCT (Reichert PT100) showed a mean difference of –0.3 mmHg, with LoA ranging from −7.3 to 6.7 mmHg. Tonnu et al. [20] also found that the Canon TX-10 NCT had a mean difference of 0.7 mmHg and LoA of ±4.8 mmHg when compared with GAT. Cook et al. [21], in a meta-analysis of eight tonometers, found that NCT devices exhibited the least disagreement with GAT, a finding similar to Patel et al.’s [11], with an average mean difference of 0.2 mmHg and LoA from –3.8 to 4.3 mmHg. Our study demonstrated comparable agreement between TRK-3 and GAT, with an overall mean difference of −0.2 mmHg and LoA ranging from −5.0 to 4.6 mmHg. These findings are consistent with previously reported NCT-GAT comparisons, suggesting that NCTs like TRK-3 generally have good agreement with GAT.

Although the overall agreement between NCTs and GAT is generally good, systematic biases have been identified and may vary with IOP levels. A previous study reported that the Reichert XPERT NCT underestimated IOP compared to GAT by 0.92 mmHg [22]. Tonnu et al. [20] observed that Cannon TX-10 NCT tended to overestimate IOP at higher values and underestimated it at lower values, which contrasts with reports suggesting that NCTs tend to overestimate IOP at low pressures and underestimate it at high pressures relative to GAT [23,24]. The latter trend is consistent with the findings of the present study, where TRK-3 OMNIA overestimated IOP in the low IOP group (mean difference: 2.1 mmHg, LoA: –1.2 to 5.4 mmHg) and underestimated IOP in the high IOP group (mean difference: –2.4 mmHg, LoA: –5.9 to 1.1 mmHg). These divergent trends may be related to factors such as differences in air-puff calibration, sensor sensitivity, and the intrinsic limitations of NCT technology, as well as possible variations in corneal biomechanics in different IOP levels. Clinically, overestimation at low IOPs may prompt unnecessary treatment escalation, while underestimation at high IOPs could delay timely intervention. Therefore, understanding these differences relative to GAT as the gold standard used in most clinical validation trials is integral when managing patients.

In this study, a TOST was conducted to assess whether TRK-3 OMNIA and GAT measurements could be considered clinically equivalent within the predefined margin of ±5 mmHg, a threshold aligned with international standards for tonometry equivalence testing [16]. This approach extends beyond traditional agreement assessments, which primarily quantify bias and variability, by establishing whether differences are small enough to allow interchangeability in clinical practice [25]. By demonstrating that the 90% CI for the mean differences in each IOP subgroup remained fully contained within this margin, this study provides statistical confirmation that TRK-3 OMNIA and GAT measurements are clinically equivalent across the tested range. The use of a 90% CI reflects the standard TOST methodology, which involves two one-sided tests, each conducted at a 5% significance level. In previous studies, stricter predefined error margins (e.g., ±2, ±3, ±4 mmHg) have been used to assess the proportion of eyes whose IOP differences fall within these thresholds [19,26]. While this approach provides a percentage-based descriptive measure of agreement, it differs from TOST, which offers a statistical confirmation of clinical equivalence. If a stricter margin were applied in this study, the 90% CI for the mean differences would still fall within a margin as narrow as ±3 mmHg across all IOP groups, indicating that TRK-3 measurements remain statistically interchangeable with GAT even under more conservative equivalence criteria. However, direct substitution without adjustment may not always be appropriate, particularly when strict IOP monitoring is required. This may highlight a need for conversion models to enhance consistency between TRK-3 OMNIA and GAT-derived IOP values.

An excellent correlation between the NCT and GAT (correlation coefficient ≥ 0.9) was previously reported [11,27]; similarly, this study found a correlation coefficient of 0.92 between TRK-3 OMNIA and GAT. The strong correlation not only further supports the potential interchangeability of TRK-3 OMNIA with GAT but also provides a foundation for conversion between the two measurement methods. Building on the strong correlation between TRK-3 OMNIA and GAT, this study established a conversion model to facilitate their interchangeability in clinical practice. The coefficient of determination was 0.84, indicating that 84% of the variance in one measurement could be explained by the other. This high predictive accuracy suggests that TRK-3 OMNIA values can be potentially converted to GAT equivalent measurements and vice versa, allowing for flexible clinical application based on the available device. Previous studies have also developed regression-based conversion formulas for specific devices and GAT [12,26,28]. However, validation of the formulas is essential for integrating them into clinical workflows. One study [19] reported that the variability of the NCT (Reichert PT100) measurements increased with higher IOP values, which is similar to our finding that the CV% of the high IOP group was the highest (6.25%), indicating that conversion performance may vary across different IOP ranges. Given the systematic trend in measurement differences, predicting one from the other is not straightforward and may require a correction model. Future work needs to refine and fully validate the conversion models in independent datasets, particularly across diverse patient populations and clinical settings, to confirm their generalizability. Additionally, nonlinear models or adaptive corrections could be explored to further optimize TRK-3 OMNIA’s conversion accuracy. However, the interpretation of TRK-3 results should be tailored to the clinical context. In high-risk settings where precise IOP readings are critical, such as postoperative management, confirmatory measurement with GAT may be warranted to avoid unintended clinical consequences.

NCT measurements have consistently demonstrated good repeatability across various devices. Previous studies reported excellent ICC of 0.91 for the Topcon CT-1P [29], 0.94 for the Oculus Corvis ST [30], and >0.9 for the Nidek Tonoref [13], while Wang et al. [14] reported a lower ICC of 0.79 for the Ocular Response Analyzer, which was still considered good and comparable to GAT. In this study, the TRK-3 OMNIA demonstrated an overall ICC of 0.94, indicating excellent repeatability, consistent with prior findings for NCT devices. When stratified by IOP levels, the high IOP group exhibited the highest ICC despite having the largest repeatability limit (4.19 mmHg) and CV (6.25%), suggesting that while absolute measurement variability increased, the relative ranking of subjects remained stable. Conversely, the intermediate IOP group had the lowest ICC, likely due to overlapping IOP values across subjects, which reduced between-subject variance. While ICC provides a measure of consistency, the repeatability limit and CV% offer a more clinically relevant interpretation for expected measurement variability. The overall repeatability limit of 3.12 mmHg and CV of 5.65% suggest that when comparing IOP measurements over time, differences exceeding these thresholds may indicate true physiological change rather than measurement variation.

This study has limitations. First, we did not account for factors like ocular surface conditions, corneal biomechanics, and refractive error, which may influence IOP measurement accuracy. Their impact on agreement and repeatability warrants further investigation. Second, while the TRK-3 OMNIA to GAT conversion formula provides an option for interchangeability, it has not been externally validated. Future studies should confirm its accuracy across diverse populations. Third, long-term reliability was not assessed, and evaluating measurement stability over time would be valuable for clinical decision-making. Addressing these limitations will enhance the clinical applicability of TRK-3 OMNIA.

## 5. Conclusions

In conclusion, this study provides strong evidence for the clinical interchangeability of TRK-3 OMNIA and GAT for IOP measurement. TRK-3 showed high agreement with GAT, with statistical equivalence across all IOP groups and a strong correlation, supporting its use as an alternative tonometry method. While systematic differences were observed at the low and high ends of the IOP spectrum, these remained within the predefined equivalence margin, and clinical discretion is advised when interpreting individual values. Additionally, TRK-3 OMNIA demonstrated good repeatability across all IOP levels, suggesting that TRK-3 OMNIA can be reliably integrated into routine clinical practice, including screening and routine follow-up assessments. Further studies are needed to assess its long-term performance and broader applicability.

## Figures and Tables

**Table 1:** Demographics of study groups.

	Low IOP Group (7 to 16 mmHg)	Intermediate IOP Group (>16 to <23 mmHg)	High IOP Group (≥23 mmHg)	All
Subject, no.	40	40	40	120
Age (years)				
Mean ± SD/Median	50.9 ± 14.7/55.0	56.0 ± 13.6/57.0	62.2 ± 11.2/65.0	56.4 ± 13.9/59.0
Age group, no. (%)				
<40 years	11 (27.5)	6 (15.0)	2 (5.0)	19 (15.8)
40–60 years	15 (37.5)	18 (45.0)	13 (32.5)	46 (38.3)
>60 years	14 (35.0)	16 (40.0)	25 (62.5)	55 (45.8)
Gender				
Male/Female	18/22	18/22	15/25	51/69
Race, no. (%)				
Caucasian	30 (75.0)	24 (60.0)	8 (20.0)	62 (51.7)
African American	3 (7.5)	9 (22.5)	30 (75.0)	42 (35.0)
Others	7 (17.5)	7 (17.5)	2 (5.0)	16 (13.3)

Abbreviations: SD, standard deviation; IOP, intraocular pressure.

**Table 2:** Intraocular pressure measurements from TRK-3 OMNIA and GAT.

	Low IOP Group (7 to 16 mmHg)	Intermediate IOP Group (>16 to <23 mmHg)	High IOP Group (≥23 mmHg)	All
TRK-3 OMNIA IOP (mmHg)				
Mean (SD)	16.6 (2.0)	19.0 (1.9)	24.3 (4.6)	19.9 (4.5)
Median	16.0	19.0	23.0	19.0
GAT IOP (mmHg)				
Mean (SD)	14.5 (1.6)	19.2 (1.6)	26.7 (4.5)	20.1 (5.8)
Median	14.0	19.0	25.0	19.0

Abbreviations: SD, standard deviation; GAT, Goldmann applanation tonometry; IOP, intraocular pressure.

**Table 3:** Repeatability of TRK-3 OMNIA intraocular pressure measurements.

	Low IOP Group (7 to 16 mmHg)	Intermediate IOP Group (>16 to <23 mmHg)	High IOP Group (≥23 mmHg)	All
Intraclass Correlation Coefficient	0.85	0.79	0.90	0.94
Repeatability Standard Deviation (mmHg)	0.80	0.94	1.51	1.13
Repeatability Limit (mmHg)	2.22	2.60	4.19	3.12
Coefficient of Variation (%)	4.82	4.94	6.25	5.65

## Data Availability

The dataset is available on request from the authors.
